# Gene therapy for children with X-linked myotubular myopathy: a plain language summary of publication for the ASPIRO study

**DOI:** 10.1177/26330040251362885

**Published:** 2025-09-18

**Authors:** Perry B. Shieh, Wendy Hughes, Marie Wood, Alan H. Beggs, Michael W. Lawlor, Julie Coats, Fatbardha Varfaj, Robert J. Graham, Nancy L. Kuntz, James J. Dowling, Wolfgang Müller-Felber, Carsten G. Bönnemann, Ana Buj Bello, Laurent Servais, Vicky MacBean, Francesco Muntoni, A. Reghan Foley, Astrid Blaschek, Emma S. James, Andreea Seferian, Lindsay N. Alfano, Tina Duong, Mojtaba Noursalehi, Weston Miller, Jun Lee, Suyash Prasad, Salvador Rico

**Affiliations:** 1 Department of Neurology, David Geffen School of Medicine at UCLA, Los Angeles, CA, USA; 2 Myotubular Trust, London, UK; 3 MTM-CNM Family Connection, Methuen, MA, USA; 4 Division of Genetics and Genomics, The Manton Center for Orphan Disease Research, Boston Children’s Hospital, Harvard Medical School, Boston, MA, USA; 5 Department of Pathology and Laboratory Medicine, Medical College of Wisconsin, and Diverge Translational Science Laboratory, Milwaukee, WI, USA; 6 Astellas Gene Therapies, San Francisco, CA, USA; 7 Division of Critical Care Medicine, Boston Children’s Hospital, Harvard Medical School, Boston, MA, USA; 8 Division of Neurology, Ann & Robert H Lurie Children’s Hospital of Chicago, Chicago, IL, USA; 9 Division of Neurology, The Hospital for Sick Children, Toronto, ON, Canada; 10 Department of Paediatric Neurology and Developmental Medicine, Hauner Children’s Hospital, Ludwig Maximilian University of Munich, Munich, Germany; 11 Neuromuscular and Neurogenetic Disorders of Childhood Section, NINDS, NIH, Bethesda, MD, USA; 12 Généthon, Evry, France, and Université Paris-Saclay/Université Evry, INSERM, Généthon, INTEGRARE Research Unit UMR_S951, Evry, France; 13 Department of Paediatrics, MDUK Oxford Neuromuscular Centre and NIHR Oxford Biomedical Research Centre, University of Oxford, Oxford, UK; 14 Department of Health Sciences, Brunel University of London, London, UK; 15 NIHR, Great Ormond Street Hospital, Biomedical Research Centre, University College London Institute of Child Health, London, UK; 16 I-Motion, Hôpital Armand Trousseau, Paris, France; 17 Abigail Wexner Research Institute, Nationwide Children’s Hospital, Columbus, OH, USA; 18 Department of Neurology, Stanford University, Palo Alto, CA, USA

**Keywords:** gene therapy, resamirigene bilparvovec, X-linked **myotubular myopathy**

## Abstract

What is this summary about?

This summary describes the results of a research study (clinical trial) called ASPIRO that was published in the *Lancet Neurology* in 2023. This study looked at an investigational gene therapy called **resamirigene bilparvovec** (also known as AT132) as a possible treatment for children with a disease called X-linked **myotubular myopathy** (abbreviated as XLMTM).

**Click here to view the PDF**

## Declarations

**Ethics approval and consent to participate:** The trial design was reviewed and approved by the institutional review boards of all institutions that took part in the trial. During the design of the study, patient and family meetings were held with the investigators to better understand the human impact of the disease and to help choose the most relevant endpoints to measure in the trial. An independent data and safety monitoring committee monitored the integrity and safety of the trial. Written informed consent to participate was given by the parents or legal guardians of the children in the ASPIRO and INCEPTUS trials.

**Availability of data and materials:** Details for how researchers may request access to anonymized participant level data, trial-level data, and protocols from Astellas sponsored clinical trials can be found at https://www.clinicaltrials.astellas.com/transparency/.

